# The spatial architecture of neuroimmune interactions in epilepsy

**DOI:** 10.3389/fimmu.2026.1742927

**Published:** 2026-02-16

**Authors:** Dianwu Chu, Wuhao Zhang, Xing Zhou, Hang Yin, Jian Yin

**Affiliations:** 1 Department of Neurosurgery, The Second Affiliated Hospital of Dalian Medical University, Dalian, China; 2 Institute for Computational Biomedicine, Department of Physiology and Biophysics, Weill Cornell Medicine, New York, NY, United States

**Keywords:** astrocyte, epilepsy, immune microenvironment, microglia, neuroinflammation, spatial transcriptomics

## Abstract

Epilepsy is increasingly recognized as a disorder not only of neuronal dysfunction but also of immune dysregulation within the central nervous system (CNS). Accumulating evidence points to a critical role for the immune microenvironment in shaping epileptogenesis—the process that underlies the development and progression of epilepsy. In this Review, we examine the spatial dynamics of neuroimmune interactions, highlighting how local inflammatory niches emerge and evolve across brain compartments such as the parenchyma and perivascular space. We describe how the spatial organization and activation of resident glial cells, alongside the infiltration of peripheral immune cells facilitated by blood–brain barrier (BBB) disruption, contribute to region-specific patterns of neuroinflammation. Critically, we emphasize that understanding “where” these neuroimmune interactions occur—their precise spatial organization within distinct cellular microenvironments—is as fundamental as identifying “what” immune cells are involved or “how” they function. Particular focus is given to the localized actions of immune mediators, including regulatory T cells and pro-inflammatory cytokines such as IL-1β, IL-6, and TNF-α, and their influence on neuronal excitability. We also discuss the spatiotemporal heterogeneity of immune signatures across different epilepsy syndromes, drawing from both experimental models and clinical observations. Finally, we explore emerging therapeutic strategies that target spatially defined immune responses and consider the potential of spatial biomarkers and advanced tissue-mapping technologies to refine disease classification and guide precision therapies. By positioning the spatial immune landscape as a central feature of epileptogenesis, we propose a framework for developing effective, potentially curative interventions for epilepsy.

## 1 Introduction

Epilepsy encompasses a group of neurological disorders characterized by aberrant hypersynchronous neuronal activity and a chronic predisposition to generate spontaneous epileptic seizures (1). Despite the availability of numerous anti-seizure medications (ASMs), approximately 30% of patients continue to experience seizures, highlighting the significant unmet need for more effective treatments (2). Furthermore, current neurocentric therapies are largely symptomatic, failing to address the underlying epileptogenic process that leads to the development and progression of epilepsy following an initial precipitating insult, such as brain injury, stroke, or infection. Consequently, there is a critical need to identify the fundamental biological mechanisms driving epileptogenesis to enable the development of preventative or disease-modifying therapeutic strategies (2, 3).

Over the past two decades, research has increasingly implicated the immune system and inflammatory processes within the CNS as key contributors to the pathophysiology of epilepsy (2, 4, 5). Initial observations of inflammatory mediators in surgically resected human epileptic foci and subsequent mechanistic studies in animal models have solidified the concept of neuroinflammation as more than an epiphenomenon. Immune mediators, including cytokines and danger signals released by activated glial cells or infiltrating leukocytes, can directly influence neuronal function through various mechanisms. These include altering neurotransmitter release and uptake, modulating voltage- and ligand-gated ion channels, and influencing synaptic plasticity and strength, thereby impacting neuronal excitability and network synchronization (2, 4, 6, 7). This recognition establishes neuroinflammation not merely because of seizure activity but as an active participant in the disease state.

Understanding the immune microenvironment—the complex, dynamic interplay between immune cells, glial cells, neurons, vascular components, and signaling molecules within specific tissue contexts—offers a crucial refinement to the concept of neuroinflammation in epilepsy. Hints towards its importance arise from observations that immune responses are often highly localized to specific brain regions or circuits implicated in seizure onset and propagation (8–11). Furthermore, the functional consequences of immune activation likely depend heavily on the precise cellular composition and molecular milieu within these localized niches. For instance, the balance between pro-inflammatory and regulatory immune cells, the integrity of the BBB at specific vascular segments, and the local concentration gradients of cytokines can collectively determine whether the net effect is pro-convulsant or potentially protective within a given microdomain (12–16). Deciphering these intricate local interactions is fundamental because targeting specific components of this microenvironment, rather than inducing broad immunosuppression, may offer more precise therapeutic strategies. This approach holds potential for addressing clinical challenges such as pharmaco-resistance, which might arise from persistent, localized inflammatory processes within the epileptogenic zone that are not addressed by conventional ASMs targeting neuronal mechanisms alone.

While the fundamental contribution of neuroinflammation to epileptogenesis is increasingly recognized, with numerous studies elucidating the identity (‘what’) and mechanistic functions (‘how’) of key immune cells and mediators, the critical dimension of spatial organization (‘where’) remains less systematically explored (9, 17). This Review aims to address this gap by synthesizing current knowledge specifically through a dedicated spatial lens. We focus on how the spatial distribution, microanatomical compartmentalization, and localized interactions of immune components—spanning resident glia, infiltrating leukocytes, and signaling molecules within the neural tissue—mechanistically contribute to seizure generation and the pathophysiology underlying epilepsy chronicity. Spatially resolved assays are beginning to be applied to human epileptogenic tissues; however, systematic multi-compartment maps that integrate immune phenotypes with barrier readouts and circuit features remain scarce. Furthermore, advances in 3D electron microscopy are providing unprecedented granular views of neuro-glial-vascular architectures (18–20). This review integrates spatially relevant insights gleaned from existing human epilepsy studies and common rodent models (kainic acid, pilocarpine). We aim to provide a framework underscoring the significance of location in neuroimmune events and advocate for concerted research efforts employing spatially resolved omics to advance epilepsy diagnostics and therapeutics.

## 2 Neuroglial cells: spatially dynamic responders in epilepsy

### 2.1 Astrocytes and the epileptogenic microenvironment

**Figure 1:**
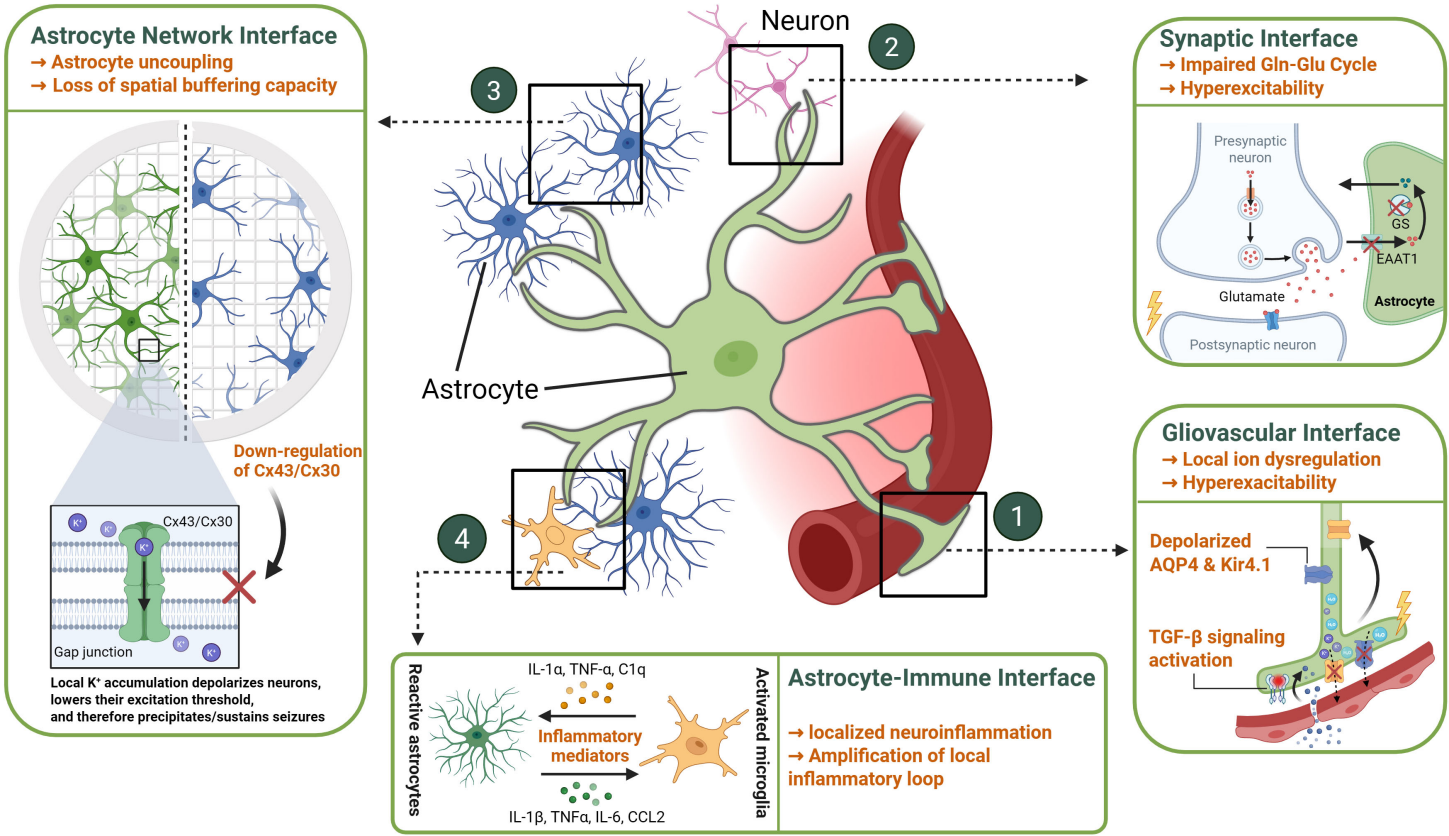
Spatially organized astrocyte dysfunction across microglia vascular, synaptic, and network interfaces during epileptogenesis. During epileptogenesis, astrocytes exhibit spatially coordinated dysfunctions across three major interfaces (1): Gliovascular interface – perivascular mislocalization of AQP4 and Kir4.1 at astrocytic endfeet leads to ionic disequilibrium, edema, and BBB leakage. Inflammatory mediators (IL-1β, TNF-α, TGF-β) exacerbate these alterations, collectively transforming astrocytic domains into pro-convulsant niches that sustain seizure activity. (2) Synaptic interface – reduced EAAT1/2 and glutamine synthetase impair glutamate clearance and GABA replenishment, promoting local hyperexcitability; (3) Astrocyte network interface – loss of gap-junction coupling (Connexin 43/30) disrupts spatial buffering of K^+^ and metabolites; (4) Microglia interface- the influence between astrocytes and microglia is bidirectional. Reactive astrocytes can release various inflammatory mediators that activate microglia, while activated microglia can in turn act upon astrocytes via specific signaling molecules. Created with BioRender (https://biorender.com/).

Astrocytes, the most numerous glial cells in the CNS, establish an intricate spatial organization, tiling the parenchyma with distinct yet interacting domains and extending elaborate processes to contact multiple cellular elements including synapses, neuronal somata, other glia, and the vasculature (21–23). This complex spatial arrangement underpins their critical homeostatic functions, but becomes profoundly disrupted during epileptogenesis, positioning astrocyte dysfunction within specific microenvironmental niches as a central element in the disease process (24–26) (**Figure 1**).

#### 2.1.1 Astrocyte spatial network

A defining spatial feature of astrocytes is their extensive interconnection via gap junctions, primarily composed of Connexin 43 (Cx43) and Connexin 30 (Cx30) (27, 28). This forms a functional syncytium, a vast cellular network crucial for spatial buffering – the rapid redistribution of ions (especially K+), water, and energy metabolites (like glucose and lactate) from areas of high concentration or demand to areas of lower concentration or supply, often directed towards blood vessels (29–32). This spatial averaging capacity is vital for maintaining ionic and metabolic homeostasis across brain regions, particularly during intense neuronal activity (31, 32). A critical pathological hallmark identified in human mesial temporal lobe epilepsy with hippocampal sclerosis is the dramatic loss of functional gap junction coupling between astrocytes, effectively dismantling the syncytium within the sclerotic region (33). Functional assays, including dye-coupling studies and electrophysiological recordings showing a loss of characteristic passive currents, confirm this uncoupling (33, 34). Crucially, studies in animal models demonstrate that this functional uncoupling is an early event in epileptogenesis, preceding significant neuronal loss and the onset of spontaneous seizures (33). This early disruption severely impairs the capacity for spatial buffering of K+ and likely glutamate within the affected tissue, directly promoting neuronal hyperexcitability and contributing causally to the epileptogenic process (30, 33, 35). Evidence strongly suggests that inflammatory signaling, potentially triggered by seizure activity or initial injury and mediated via pathways like Toll-like Receptor 4 (TLR4), drives this pathological uncoupling (33, 36). The precise spatial extent and progression of this network breakdown during epileptogenesis remain key areas for investigation.

#### 2.1.2 Astrocyte-neuron interactions: synaptic and metabolic microdomains

Astrocytes establish highly localized interactions with neurons at the tripartite synapse, where their fine processes (perisynaptic astrocytic processes) lie in proximity of nanometer scales to the pre- and post-synaptic neuronal elements (21, 37). This intimate spatial relationship allows astrocytes to exert precise control over the synaptic microenvironment. They achieve this through the rapid uptake of neurotransmitters, particularly glutamate, via transporters like EAAT1 (Excitatory Amino Acid Transporter 1) and EAAT2 (Excitatory Amino Acid Transporter 2) which are concentrated on these perisynaptic astrocytic processes (25, 36, 38). They also release gliotransmitters (e.g., glutamate, ATP/adenosine, D-serine, potentially GABA via Best1 channels) which can act locally on presynaptic or extrasynaptic neuronal receptors to modulate synaptic transmission and plasticity (23, 35, 39, 40). Furthermore, astrocytes provide spatially targeted metabolic support to active synapses. This includes the vital glutamate-glutamine cycle, where astrocyte-specific glutamine synthetase converts the uptaken glutamate to glutamine, which is then shuttled back to neurons as a precursor for both glutamate and GABA synthesis (37, 41, 42). The astrocyte-neuron lactate shuttle may also provide activity-dependent energy substrates directly to nearby neurons (22, 43). During epileptogenesis, documented reductions in EAAT expression/function and the significant loss of activity in astrocytes within epileptic foci disrupt these local interactions (40, 43, 44). This leads to glutamate accumulation in the synaptic vicinity, impaired replenishment of the GABA pool in inhibitory terminals, and potentially altered metabolic support, collectively shifting the local excitatory/inhibitory balance towards hyperexcitability (37, 40, 41). While the basic synaptic structure might remain, the functional capacity of these astrocyte-neuron interactions within the epileptic microenvironment is severely compromised (23, 25).

#### 2.1.3 Astrocyte-stromal interactions: the gliovascular interface

**Figure 2:**
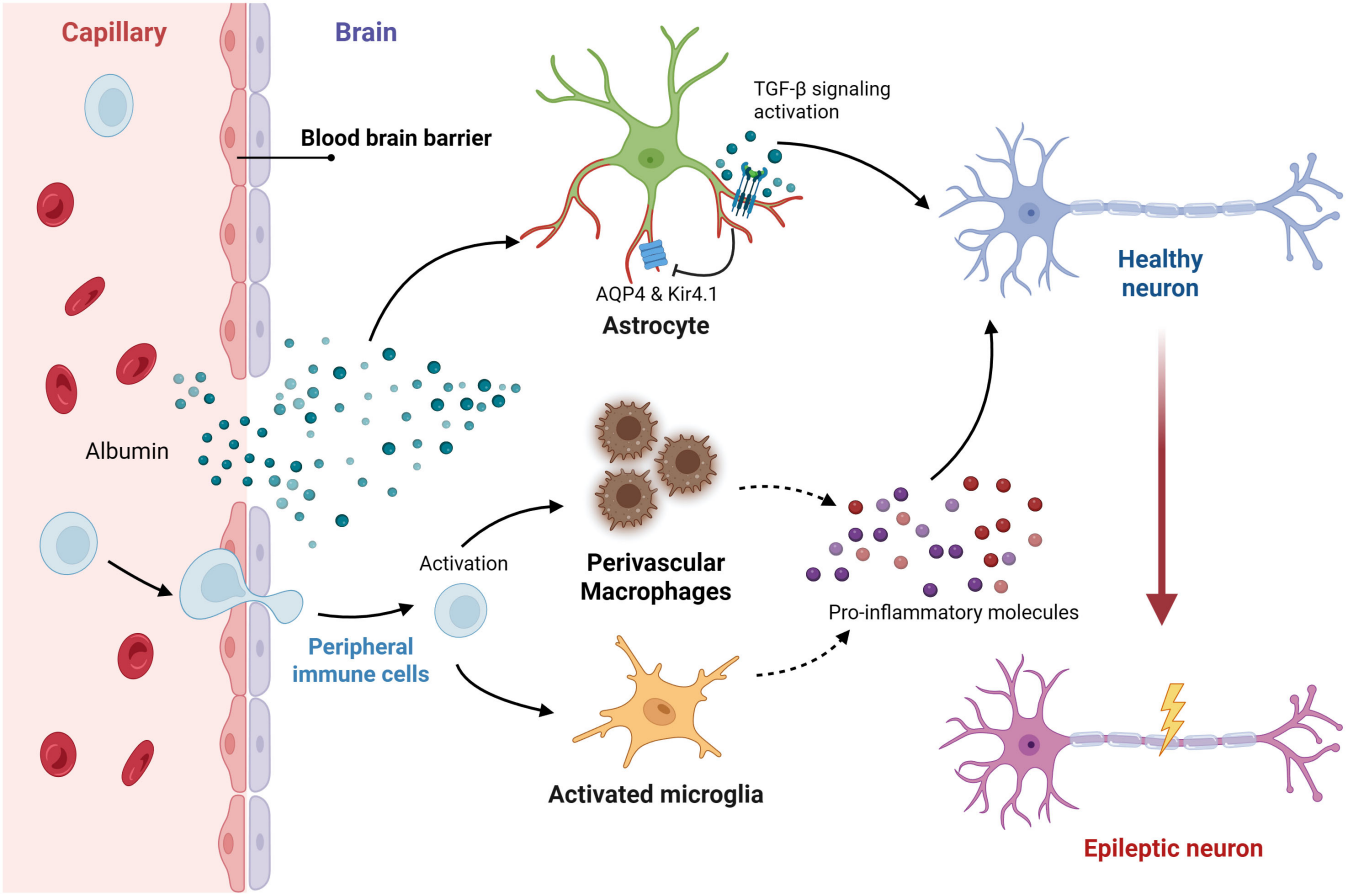
Feedback loop linking BBB disruption, astrocyte hyperactivity, and immune activation in epilepsy. Blood–brain barrier (BBB) leakage permits albumin extravasation, which activates astrocytic TGF-β signaling and disrupts AQP4 and Kir4.1 polarization at vascular endfeet. Hyperactive astrocytes release pro-inflammatory mediators that activate perivascular macrophages and microglia, which in turn amplify inflammation and further impair BBB integrity. This closed inflammatory circuit establishes a spatially confined but self-sustaining niche that drives neuronal hyperexcitability and epileptogenesis. Created with BioRender (https://biorender.com/).

The neurovascular unit represents another critical spatial interface where astrocytes play a central role. Their specialized vascular endfeet form a near-continuous sheath around brain capillaries, establishing direct contact with endothelial cells and pericytes (22, 44, 45). This anatomical arrangement is fundamental to the formation and maintenance of the BBB and the regulation of cerebral blood flow (23, 44). Key homeostatic proteins, such as the water channel AQP4 and the potassium channel Kir4.1, demonstrate a highly polarized distribution in healthy tissue, being densely concentrated specifically at these perivascular endfeet (46, 47). Epileptogenesis is frequently associated with BBB dysfunction, leading to the extravasation of serum proteins like albumin into the perivascular space (48, 49). This event specifically targets perivascular astrocytes, activating signaling pathways such as the TGF-β cascade (50). A major consequence is the downregulation and/or mislocalization of AQP4 and Kir4.1, stripping them from the endfoot membrane (51, 52). This loss of polarized channel expression at the vascular interface severely impairs the efficient siphoning of K+ and water between the brain parenchyma and the bloodstream, contributing to local ion dysregulation, failure to resolve edema, and originating hyperexcitability from this specific compartment (50, 53). This process establishes a self-sustaining closed inflammatory circuit at the gliovascular interface (**Figure 2**). Initial BBB leakage allows albumin to activate astrocytic TGF-β signaling, leading to the loss of AQP4 and Kir4.1 polarity. These dysfunctional astrocytes then release mediators that activate perivascular macrophages and microglia, which in turn secrete molecules that further compromise the barrier integrity, locking the microenvironment into a chronic epileptogenic state. Furthermore, these reactive perivascular astrocytes become localized sources of inflammatory mediators (e.g., VEGF, cytokines) and matrix-degrading enzymes, which can further compromise BBB integrity and propagate inflammation into the adjacent tissue (23, 34, 45).

#### 2.1.4 Astrocyte-immune interactions: localized neuroinflammation

Astrocytes are key orchestrators and responders within the complex inflammatory milieu of the epileptic brain, engaging in spatially defined interactions with microglia and influencing broader immune responses, particularly after BBB compromise (23, 34, 54). Reactive astrocytes release a diverse array of inflammatory mediators, including cytokines (IL-1β, TNFα, IL-6) and chemokines (CCL2), which act locally to modulate neuronal excitability, increase vascular permeability, and influence the behavior of other immune cells within their immediate vicinity (55, 56). This communication is strongly bidirectional with microglia, the brain’s resident myeloid cells (57). Microglia often become activated early following a seizure or insult and release factors (e.g., IL-1α, TNF-α, C1q) that directly influence the phenotype and function of nearby astrocytes, often promoting a pro-inflammatory or detrimental state (34, 58, 59). Studies suggest this spatially and temporally coordinated glial crosstalk is critical, with early microglial signals potentially driving subsequent pathological astrocyte changes (like uncoupling or impaired K+ buffering) that contribute to chronic hyperexcitability (33, 60). Danger signals released from damaged cells, such as High mobility group box 1 (HMGB1), further amplify this local inflammatory loop by activating TLR4 receptors expressed on both astrocytes and microglia within the affected zone (61). This localized inflammation is tightly coupled with structural remodeling of the microenvironment. Astrocyte hypertrophy during reactive gliosis physically alters the extracellular space geometry and synaptic arrangements (24). Astrocyte-derived factors, such as matrix metalloproteinases, contribute locally to extracellular matrix degradation and reorganization, while other signals influence pathological synaptic plasticity and the integrity of perineuronal nets, often showing specific vulnerability around inhibitory neurons within epileptic foci (62, 63). It is crucial to recognize the spatial heterogeneity of this response; astrocytes within the same epileptic region can adopt diverse reactive phenotypes (ranging from pro-inflammatory to potentially neuroprotective or metabolically stressed) depending on the specific local signaling cues, proximity to the lesion core, vascular structures, or specific neuronal populations (64). The precise spatial patterning of these different astrocyte states and their interactions with immune cells likely dictates the overall tissue response and contributes significantly to disease progression or potential repair mechanisms.

### 2.2 Microglia and the epileptogenic microenvironment

Microglia, the resident myeloid cells of the CNS, are not static sentinels but dynamically survey their allocated parenchymal territories via constant motile processes (65, 66). In the context of epilepsy, their activation state, spatial distribution, and function become profoundly altered, varying significantly depending on proximity to the epileptic focus, the specific brain region, and the stage of epileptogenesis (67–70). This spatial heterogeneity underscores the importance of the local microenvironment in dictating microglial roles, positioning them as critical modulators and potential drivers of pathological processes.

#### 2.2.1 Microglia spatial network

The normally organized tiling of microglia across the brain parenchyma is disrupted during epileptogenesis. Evidence from human tissue and animal models shows reactive microglia clustering near sites of neuronal injury or hyperexcitability, exhibiting hypertrophic or amoeboid morphologies distinct from their ramified resting state (67, 71, 72). This involves not only morphological transformation but also significant proliferation within these foci, increasing local microglial density (72, 73). Furthermore, microglia can pathologically infiltrate neuronal layers, such as the hippocampal pyramidal and granular layers, altering the typical cellular architecture and establishing novel spatial relationships with neuronal somata (72). This spatial reorganization implies a shift from broad surveillance to focused, potentially pathological, activity within specific microdomains. Intriguingly, studies activating Mammalian target of rapamycin (mTOR) signaling specifically in microglia demonstrate that these non-inflammatory morphological and proliferative changes alone are sufficient to drive spontaneous seizure development, highlighting how altered spatial distribution and cellular state, independent of overt inflammation, can be epileptogenic (72).

#### 2.2.2 Microglia-neuron interaction: modulating the synaptic milieu

**Figure 3:**
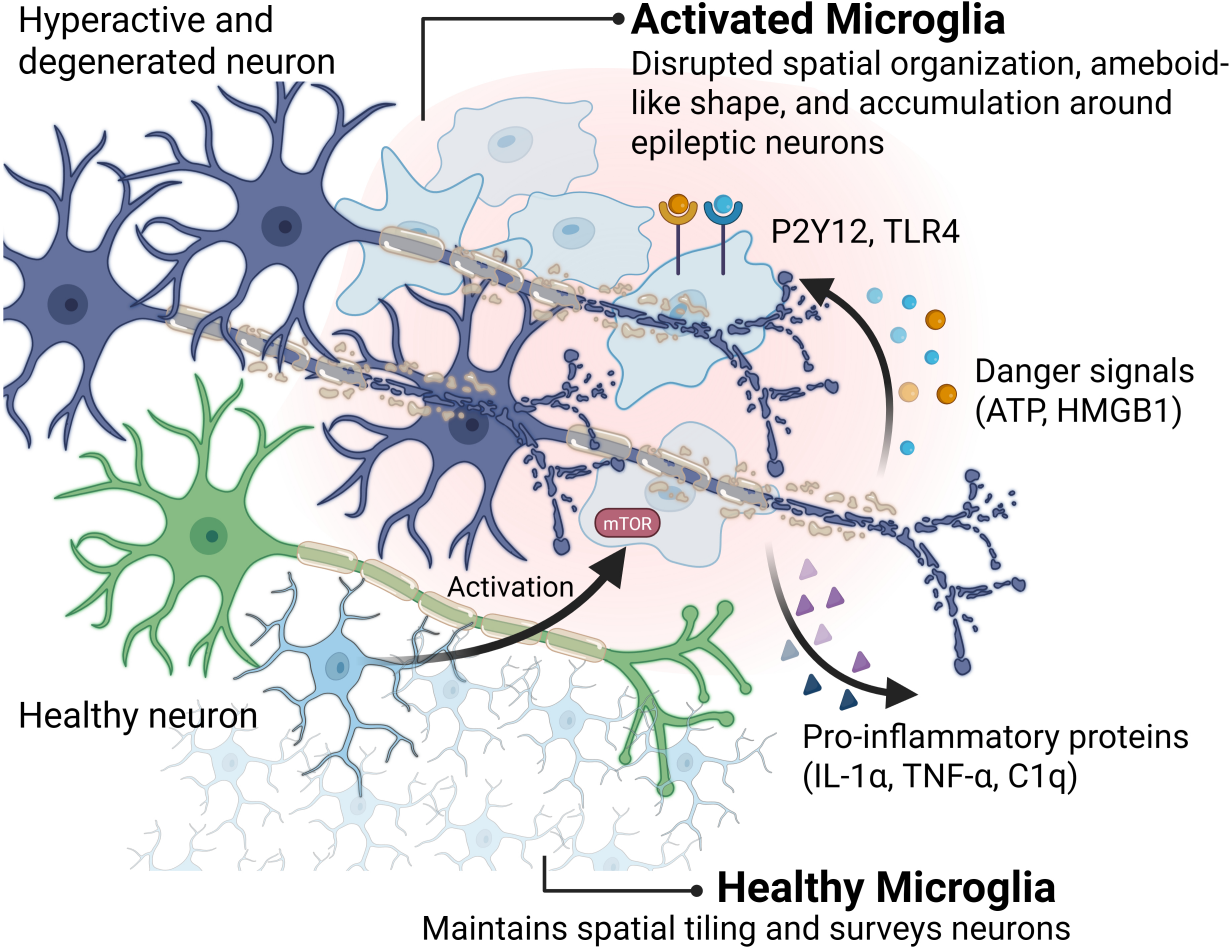
Spatial activation of microglia and the intracellular mTOR signaling hub in epileptogenic tissue. The transition from a ramified homeostatic state (green) to an amoeboid activated phenotype (blue/light blue) is triggered by danger signals (ATP, HMGB1) released from hyperactive or degenerated neurons. These signals bind to P2Y12 and TLR4 receptors, respectively, converging on the mTOR (mammalian target of rapamycin) pathway—a key intracellular driver of microglial activation. Once activated, microglia secrete pro-inflammatory proteins (IL-1α, TNF-α, C1q) that amplify local neuroinflammation. Dashed arrows indicate the recruitment of microglial processes toward injured neuronal sites. Created with BioRender (https://biorender.com/).

Microglial communication with neurons relies heavily on spatially restricted signaling and direct physical contact (**Figure 3**). Following seizure activity, localized ATP release from hyperactive neurons acts as a chemoattractant, guiding microglial processes via Purinergic receptor P2Y12 (P2Y12) receptors towards the source for spatially precise interactions (74–76). Microglia possess surface ectonucleotidases (i.e., CD39) that rapidly convert this local ATP/ADP surge into inhibitory adenosine within the immediate vicinity, acting on nearby neuronal A1 receptors as a spatially confined negative feedback loop essential for dampening excitability; disrupting this pathway worsens seizures (77, 78). In parallel, neuron-derived signals like fractalkine (CX3CL1) engage microglial CX3CR1 receptors locally, influencing the activation state and interaction dynamics specifically at that neuron-glia interface (74, 79). Direct physical contacts, including specialized somatic junctions and dendritic envelopment by “process pouches,” allow microglia to directly modulate neuronal excitability or aid structural repair within specific subcellular compartments (80). Microglia also interact intimately with the synaptic microenvironment, contributing to circuit refinement through synaptic pruning, often involving localized recognition of tags like complement components (e.g., C1q, C3) deposited on specific synapses (81, 82). From both kainic acid rodent models and resected human temporal lobe epilepsy tissue, where complement pathways are often upregulated locally (83, 84), microglia cells are reported to contribute to pathological circuit alterations through aberrant, spatially targeted synaptic elimination (67, 82). Altered microglial phagocytic capacity within the focus impacts debris clearance and inflammation (85), with enhanced phagocytosis potentially leading to excessive removal of neuronal elements (72). Local immune tone is further regulated by spatially restricted checkpoint interactions (e.g., involving neuronal CD200 or CD47 engaging microglial receptors) (67, 86), and the loss of these “calming” signals within epileptogenic lesions likely contributes to spatially persistent microglial activation (86). Thus, through dynamic spatial positioning, targeted contact, localized signaling, and influence over the synaptic milieu, microglia critically regulate the neuronal microenvironment in epilepsy. The heterogeneity of these responses across the epileptic landscape highlights the need for high-resolution spatial mapping to decipher their precise contributions and guide therapeutic development (87). The activation of microglia is a multi-step spatial process. As illustrated in **Figure 3**, danger signals like ATP and HMGB1 engage P2Y12 and TLR4 receptors, respectively, triggering the mTOR signaling pathway. This metabolic and signaling shift drives the morphological transition to an amoeboid state and culminates in the targeted secretion of pro-inflammatory proteins, including IL-1α, TNF-α, and C1q, which directly modulate the peri-neuronal environment.

Beyond these extracellular signaling pathways, converging evidence now implicates intracellular innate immune machinery within microglia as a direct driver of epileptogenic circuit remodeling. For instance, the NLRP3 inflammasome has emerged as a central molecular driver of epileptogenic inflammation. In the amygdala kindling rat model, microglial NLRP3 activation leads to the processing and release of IL-1β, amplifying local neuroinflammation. Crucially, knockdown of NLRP3 significantly suppresses the development of spontaneous recurrent seizures and reduces hippocampal neuronal loss in this model, linking intracellular immune machinery directly to circuit hyperexcitability (88–90). Moreover, recent spatial mapping has also revealed a specific vulnerability of inhibitory circuits. GABA released by stressed inhibitory neurons can act as a chemotactic ‘find-me’ signal, recruiting microglia to the inhibitory synapse. This interaction often results in the preferential engulfment of inhibitory terminals, structurally dismantling the ‘brakes’ of the circuit and driving the network toward hypersynchrony (91, 92). Exploring how microglia interact with inhibitory circuits is a promising new direction for epilepsy research.

### 2.3 Oligodendrocytes and myelin integrity

Besides the well-studied astrocytes and microglia, oligodendrocytes are vulnerable components of the spatial immune niche during epilepsy. In lithium-pilocarpine models, hippocampal demyelination and mature oligodendrocyte loss initiate during the acute phase and progressively worsen throughout epileptogenesis. Intriguingly, this degeneration triggers a compensatory, yet transient, regenerative response: oligodendrocyte precursor cells proliferate and upregulate the key differentiation factors Olig1 and Olig2 during the acute and latent windows (93). However, this repair mechanism collapses in the chronic phase as Olig1/2 expression plummets, suggesting that the persistent inflammatory microenvironment eventually overwhelms the lineage’s reparative capacity, cementing white matter injury as a permanent driver of circuit instability.

## 3 Peripheral immune cell infiltration in epilepsy

Peripheral entry into the epileptic brain follows anatomical lines of least resistance. It begins at vascular interfaces—the endothelium and Virchow–Robin (perivascular) spaces—and, from there, resolves into compact parenchymal hotspots where immune cues, glial state, and neuronal excitability become locally intertwined. Border-associated macrophages, especially perivascular macrophages, sit directly on these gateways and help set the chemokine and antigen-presentation landscape that guides leukocytes from the lumen into perivascular sleeves and on into adjacent tissue (12, 94) (**Figure 2**). Within the tissue, infiltrating lymphoid and myeloid cells do not simply add “more inflammation”: they organize around vasculature and microglial clusters, interact with astrocytic endfeet, and, in doing so, create microdomains where synaptic and ionic homeostasis fail. These spatially restricted interactions—rather than global cytokine levels—best explain why some cortical or hippocampal regions become epileptogenic while neighboring areas remain relatively quiescent.

### 3.1 Infiltrating monocytes and macrophages

Among myeloid cells, CCR2^+^ inflammatory monocytes are the most consistently implicated in seizure-linked neuroinflammation. After status epilepticus, CCL2–CCR2 signaling drives robust recruitment of blood-borne monocytes that interdigitate with activated microglia around vessels and within hippocampal laminae; genetic or pharmacologic interruption of this axis mitigates neuronal loss and downstream behavioral sequelae (95–97). Notably, the administration of small-molecule CCR2 antagonists (e.g., RS504393, RS102895) or CCL2 synthesis inhibitors (e.g., bindarit) effectively blocks the ingress of blood-borne monocytes (97, 98). In kainic acid models, these interventions interrupt the downstream Signal Transducer and Activator of Transcription 3 (STAT3)/IL-1β signaling cascade, significantly reducing hippocampal neuronal death and attenuating the severity of spontaneous recurrent seizures (97). Furthermore, recent findings identify a critical therapeutic window where brief CCR2 antagonism post-status epilepticus prevents long-term cognitive comorbidities; this “fleeting” inhibition is sufficient to rescue deficits in working and retention memory and preserve BBB integrity (98, 99). While the impact on seizure arrest can be model-dependent, with studies in pilocarpine rats demonstrating neuroprotection and CA1 volume preservation without a reduction in spontaneous seizure frequency (100), the consensus across models highlights that targeting monocyte infiltration effectively creates a neuroprotective environment and disrupts the inflammatory maintenance of the epileptogenic niche. This vascular interface serves as a critical spatial niche where infiltrating monocytes integrate into a localized neuro-glial signaling loop. Perivascular macrophages at these entry sites not only secrete CCL2 and present antigens but also respond to astrocyte-derived CCL2, collectively enhancing monocyte recruitment and modulating T-cell responses. This activity further aggravates BBB disruption, facilitating increased infiltration of peripheral immune cells. These functions that underscore how “where” these cells act (at the vascular border) is inseparable from “what” they do (amplify leukocyte in-flux and antigen surveillance) (12).

Two mechanistic threads are worth emphasizing for an epileptogenesis framework. First, monocyte-derived macrophages sustain IL-1β/STAT3 signaling cascades that degrade astrocytic K^+^/glutamate buffering and weaken inhibitory drive, embedding hyperexcitability into local circuits (97). Second, border macrophages can loosen BBB properties and remodel the perivascular niche, further lowering the threshold for repeated waves of leukocyte entry during recurrent seizures (12, 101). These data argue for compartment-directed interventions—e.g., CCR2 antagonism or perivascular macrophage modulation—as disease-modifying strategies aimed at the gateways that nucleate pro-convulsant microenvironments.

### 3.2 T Lymphocytes in the epileptic microenvironment

T cells also accumulate in specific places and windows, and their programs map onto those spaces. In pediatric drug-resistant focal epilepsies, specifically Type II Focal Cortical Dysplasia, resected lesion centers from the frontal and temporal lobes contain abundant memory CD4^+^ and CD8^+^ T cells together with γδ T cells that produce IL-17 and GM-CSF; by contrast, FoxP3^+^ regulatory T cells (Tregs) are present but tend to track inversely with inflammatory burden. Functionally, IL-17 and GM-CSF from γδ cells increase neuronal firing and compromise neuronal viability in slice preparations, while the Treg compartment restrains this inflammatory tone—an arrangement that captures, within a single lesion, how effector and regulatory T cells can pull in opposite directions (102). Human temporal lobe epilepsy associated with glutamic acid decarboxylase (GAD) antibodies adds a temporal dimension: early “encephalitic” phases show dense parenchymal and perivascular lymphocytic cuffs, dominated by granzyme-B^+^ CD8^+^ cytotoxic T cells and plasma cells in hippocampus; later “low-activity” phases exhibit a thinner immune presence despite ongoing clinical disease (103). The causal link from CD8^+^ attack to epilepsy is supported by a neuron-targeted limbic encephalitis model in which antigen expression confined to CA1 leads to priming of CD8^+^ T cells in deep cervical lymph nodes, a wave of hippocampus-restricted CD8^+^ infiltration within the first week, albumin leakage at the BBB, acute seizures and memory impairment, and, ultimately, chronic spontaneous seizures (103). Taken together, these human and mechanistic data converge on the same principle: discrete T-cell populations gather in defined hippocampal and cortical compartments and, there, set the stage for enduring epileptogenic niches (102–104).

**Figure 4:**
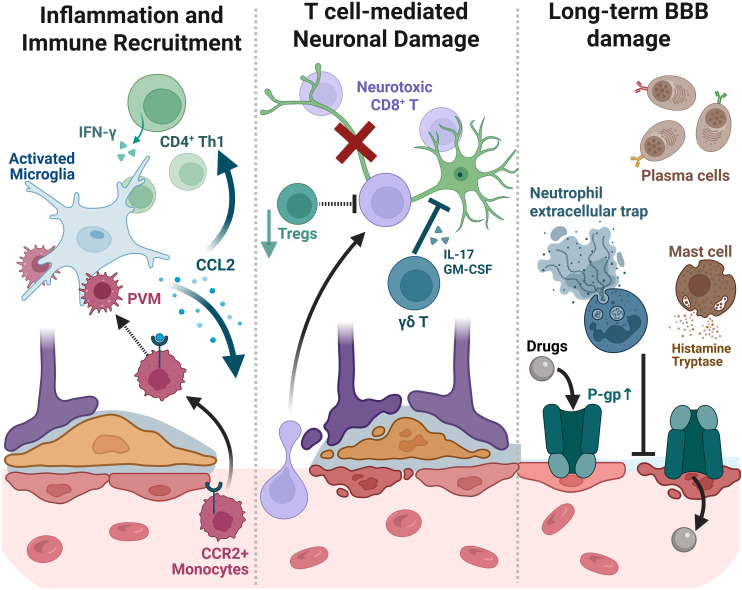
The spatial cascade of peripheral immune infiltration and the formation of chronic epileptogenic niches. The progression is depicted in three functional stages. Left (Recruitment): CCL2 and IFN-γ gradients guide the entry of CCR2^+^ monocytes and Th1-skewed CD4^+^ T cells, which interact with resident activated microglia. Middle (Damage): Infiltrating CD8^+^ cytotoxic T cells and γδ T cells execute localized neuronal attack via IL-17 and Granzyme B, while the “T” bars indicate the failing suppressive role of Tregs (regulatory T cells). Right (Chronicity & Resistance): Long-term consequences include NET (neutrophil extracellular trap) formation and mast cell-mediated release of tryptase and histamine, exacerbating barrier fragility. Critically, the upregulation of P-gp (P-glycoprotein) on microvessels (indicated by the green transporter) enhances drug efflux, contributing to pharmacoresistance. Each cell type occupies distinct microanatomical compartments as detailed in Section 3. Created with BioRender (https://biorender.com/).

CD4^+^ T cells contribute a second, clinically relevant axis. In adults with temporal lobe epilepsy, a peripheral Th1/Th2 skew (increase in IFN-γ, decrease in IL-4) is mirrored in the brain by increased CD4^+^ infiltration with frequent adjacency to Iba1^+^ microglia in hippocampus and neocortex. Mechanistically, Notch1 emerges as a regulatory switch for both Th1 differentiation and CD4^+^ trafficking across a permeable barrier; inhibiting Notch1 dampens microglial activation, reduces seizures, and improves cognition in experimental systems (105). On the regulatory side, Tregs are not merely present—they are prognostically meaningful. In patients and models, higher Treg numbers within epileptogenic zones associate with fewer seizures, whereas experimental Treg depletion worsens gliosis, cytokine production, neuronal loss, and seizure burden; conversely, expanding Tregs in the brain suppresses seizures (16). Together, these strands argue that the composition and positioning of T-cell subsets—CD8^+^ in hippocampal laminae, Th1-skewed CD4^+^ adjacent to microglia, γδ cells and Tregs competing within lesion cores—are central to how immune pressure becomes epileptogenic (16, 102, 105) (**Figure 4**).

These altered T lymphocyte balances are frequently mirrored in the periphery. Flow cytometric analyses of peripheral blood from patients with intractable epilepsy reveal a significant systemic peripheral T cell imbalance, characterized by an elevated Th17/Treg ratio. This skewing is functionally critical because the excess Th17 cells secrete IL-17A, which disrupts BBB integrity, and simultaneously, the reduction in Tregs diminishes the systemic ‘braking’ of inflammation (106, 107). Notably, this ratio is dynamic; therapeutic interventions such as the ketogenic diet have been shown to restore the Th17/Treg balance towards a healthy baseline, correlating with reduced seizure frequency (107).

### 3.3 Immune-glia crosstalk and hyperexcitability

At juxtavascular sites, endothelial cells and perivascular macrophages concentrate chemokines and present antigen, drawing T cells and CCR2^+^ monocytes into perivascular sleeves that lie against astrocytic endfeet and microglial processes (12) (**Figure 4**). Within these sleeves and the adjacent parenchyma, CD8^+^ T cells form synapses with MHC-I–positive neurons and execute cytotoxic programs; in GAD-antibody temporal lobe epilepsy, neuronal death is predominantly T-cell mediated, not complement-driven (103). Th1-skewed CD4^+^ cells, frequently *in situ* neighbors of microglia, license microglial and monocyte-derived macrophage responses through IFN-γ and TNF, amplifying IL-1 family signaling and pushing inhibitory–excitatory balance toward hypersynchrony. Meanwhile, CCR2-dependent monocyte influx reinforces this state through STAT3-linked IL-1β production that erodes local astrocytic and synaptic homeostasis (96, 97). In short, infiltrating lymphoid and myeloid cells *organize* pro-convulsant glial states at vascular and synaptic borders. That organization—and its tight spatial footprint—helps explain why small portions of hippocampus or neocortex can become durable seizure generators even as neighboring tissue remains comparatively unaffected.

Additionally, some often-overlooked contributors deserve mention in this spatial frame, which are the B cells, neutrophils, and mast cells. For B-lineage cells, the clearest human signal remains plasma-cell-rich early stages of GAD-antibody temporal lobe epilepsy; although antibodies may be bystanders to CD8-mediated injury, their presence delineates an active, perivascular/parenchymal niche and—importantly—marks a window where immunotherapy can still modify trajectory (103). While B cells have received less attention than their T-cell counterparts, they likely play a multifaceted role in the epileptogenic niche that extends beyond systemic autoantibody production. In addition to differentiating into antibody-secreting plasma cells—which cluster in perivascular spaces during the early active phases of autoimmune epilepsies—B cells can function as potent antigen-presenting cells to CD4+ T cells (108). Furthermore, they serve as a localized source of pro-inflammatory cytokines, including IL-6, GM-CSF, and TNF-α. This allows B cells to sustain local inflammatory loops and promote BBB permeability even in the absence of specific neuronal autoantibodies, acting as ‘architects’ of the chronic immune microenvironment rather than mere transient visitors. Next, neutrophils adhere to cerebral microvessels after seizures through LFA-1/ICAM-1 interactions and prolong postictal hypoperfusion. Blocking neutrophil adhesion normalizes cerebral blood flow, implicating early neutrophil–endothelial dynamics in vascular dysfunction at seizure foci (109). Emerging work on neutrophil extracellular traps suggests they aggravate BBB permeability in CNS disease; experimental NET inhibition protects barrier function and cognition, raising a plausible link to seizure-provoked BBB fragility that merits direct testing in epilepsy (110, 111). Finally, mast cells, which are the early vascular sentinels, can loosen the BBB via histamine and tryptase; while epilepsy-specific evidence is still limited, seizure-linked histamine release and benefit from mast-cell stabilization in experimental status suggest a small but potentially catalytic role at the breach (112, 113). That organization—and its tight spatial footprint—helps explain why small portions of hippocampus or neocortex can become durable seizure generators even as neighboring tissue remains comparatively unaffected (**Figure 4**, left and middle panels).

### 3.4 Long-term consequences

A critical question is whether persistent inflammatory niches contribute to epileptogenesis and pharmaco-resistance when immune resolution fails. Evidence suggests indeed they do. Chronically epileptic regions often show enduring, focal “smoldering” inflammation with microglial activation, gliosis, elevated cytokines, and ongoing trafficking of peripheral cells (114). In cohorts with epilepsy of unknown etiology, neural autoantibody prevalence is ~7–8% on average, with some series reporting up to ~15–20% depending on inclusion criteria, supporting an occult immunologic contribution in a subset rather than one-third across the board (115, 116). One consequence is BBB transporter remodeling that undermines antiepileptic drug efficacy. HMGB1, released by neurons and glia under chronic seizure stress—induces BBB P-glycoprotein via TLR4/RAGE-NF-κB signaling, enhancing drug efflux and lowering intraparenchymal drug levels (117, 118). This pathological state is then ‘locked in’ by short-range feedback loops, including the upregulation of multidrug transporters like P-glycoprotein, which physically and functionally reinforce the inflammatory architecture (**Figure 4**, right panel). Consistently, refractory patients and models show pro-inflammatory cytokine upregulation accompanied by multidrug transporter increases. Notably, COX-2 inhibition can restore phenobarbital anticonvulsant efficacy in a rat model of pharmacoresistant temporal lobe epilepsy, illustrating a direct link between unchecked inflammation and drug resistance (119). Conversely, enhancing immune resolution—e.g., boosting Tregs or recruiting them to foci via CCL20—dampens neuroinflammation and reduces seizures *in vivo* (16). Taken together, these data support immunomodulatory strategies (targeting CCR2^+^ monocytes, pro-inflammatory T cells, or augmenting Tregs) as disease-modifying adjuncts aimed at dismantling the inflammatory architecture that supports chronic seizures.

Besides, in cohorts with epilepsy of unknown etiology, neural autoantibody prevalence is ~7–8% on average, supporting an occult immunologic contribution in a subset rather than one-third across the board. Within this seropositive population, the pathogenic mechanisms vary significantly. While the frequently identified GAD65 antibodies typically serve as markers for T-cell mediated cytotoxicity, other autoantibodies directly disrupt synaptic function. Specifically, antibodies targeting the glycine receptor have been identified in patients with progressive epilepsy variants that are notably responsive to immunotherapy (120, 121). Mechanistically, these antibodies exert a direct pathogenic effect: *in vitro* studies using cultured neurons demonstrate that they can directly antagonize glycine receptor currents and induce receptor internalization, thereby disinhibiting neural circuits and promoting hyperexcitability. This highlights a direct functional link where the immune effector (the antibody) physically obstructs the molecular machinery of inhibition (122).

## 4 Discussion and prospects

### 4.1 Discussion

In this review, we advance a spatial thesis of epileptogenesis: seizures emerge and persist because small, anatomically constrained inflammatory niches destabilize circuit homeostasis. We argue that in epilepsy, “where” interactions occur conditions both “what” cells are present and “how” they communicate. Evidence across models and human tissue aligns with a cascade in which focal BBB leakage seeds a perivascular niche; albumin/TGF-β signaling at astrocytic endfeet disrupts Aquaporin-4 (AQP4)/Inwardly rectifying potassium channel 4.1 (Kir4.1) polarity and gap-junction coupling; microglia re-cluster around stressed synapses and altered extracellular matrix; and recruited leukocytes reinforce the same neighborhoods through cytokines and matrix metalloproteinases. These events co-localize in perivascular sleeves, lesion rims, and layer-specific strata, generating self-reinforcing microdomains of local neuroinflammation and neuronal hyperexcitability.

A spatial lens helps reconcile why seemingly “diffuse” neuroinflammation yields focal seizure onset zones. Astrocytes that are well-coupled can dissipate ionic and metabolic burden over distance; when their network fragments, the same burden accumulates locally and tips neurons toward pathological firing. Microglia, guided by ATP and chemokine gradients, concentrate where astrocytic polarity and synaptic support have faltered, shifting from homeostatic surveillance to short-range modulation of synapses and perineuronal nets. Peripheral cells do not simply flood the parenchyma; their ingress is anatomically gated at leaky microvessels, and their effector functions—proteolysis, cytokine release, antigen presentation—remain most active near those gates. The result is not global immune activation but small neighborhoods with the right geometry and cell mix to sustain excitability.

This framework also recontextualizes pharmacoresistance as a spatial mismatch between therapy and pathology. While ASMs effectively reduce neuronal excitability, they typically fail to address the underlying structural deficits—such as BBB leakage, astrocyte uncoupling, and extracellular matrix degradation—that allow hyperexcitable conditions to regenerate once the drug’s effect subsides. Drug exposure itself may be spatially heterogeneous, as lesion rims can express efflux transporters or harbor diffusion barriers, and an edematous or fibrotic matrix can alter local tissue pharmacokinetics. This pathological state is then “locked in” by short-range feedback loops, including dysregulated purinergic signaling, complement-mediated tagging of synapses, and TGF-β autocrine signaling in perivascular astrocytes. These observations strongly suggest that a combination therapy—pairing a conventional ASM with an intervention aimed at niche repair (e.g., stabilizing gliovascular signaling, restoring astrocyte coupling, or modulating microglial checkpoints)—would be more effective than monotherapy, particularly for treating persistent focal epilepsy.

### 4.2 Prospects

First, modern spatial technologies render the hypothesis that epilepsy is a disease of inflammatory places, beyond diffuse neuroinflammation, directly testable (123). Spatial transcriptomics and multiplex imaging can simultaneously map barrier integrity, astrocyte polarity, microglial states, immune phenotypes, and the cellular interactions within a single tissue section, anchoring these molecular readouts to electrophysiological data and anatomical signatures. By carefully sampling the seizure focus, its surrounding rim, and contralateral tissue, such datasets can generate composite biomarkers—like perivascular disruption indices or astrocyte network integrity scores—that can stage disease and stratify patients. “Window-of-opportunity” clinical trials in surgical candidates are especially promising; administering a short, spatially targeted intervention before resection would allow for direct, on-tissue assessment of pharmacodynamic effects (such as restored AQP4 polarity), providing causal evidence that conventional trials often lack.

Moreover, the spatial heterogeneity of neuroinflammation offers a mechanistic rationale for the efficacy of neuromodulatory therapies such as vagus nerve stimulation, Deep Brain Stimulation, and responsive neurostimulation. Beyond their electrical effects on neuronal synchrony, these interventions may exert anti-inflammatory effects via the cholinergic anti-inflammatory pathway (VNS) or by locally modulating the glial milieu at the stimulation site (DBS). Future trials could leverage spatial biomarkers to identify patients with “inflammatory” focal architectures who might benefit most from targeted neurostimulation that doubles as immunomodulation.

Lastly, the spatial model also illuminates the roles of often-overlooked cell types. Neutrophils, appearing early after a BBB breach, can initiate proteolytic cascades that amplify leakage. Monocytes and perivascular macrophages shape the local chemokine environment and orchestrate antigen presentation at these gateways. The activity of T cell subsets can either propagate or resolve inflammation depending on local cues, while B cells and plasma cells may contribute regionally through antibody and cytokine production. These contributions are unlikely to be uniform; instead, they probably reflect the specific architecture and vascular supply of a given brain region, helping to explain why diverse etiologies like developmental lesions, post-infectious scarring, and autoimmune encephalitis can converge on similar clinical phenotypes.

However, it should be noted that important limitations remain. Spatial assays often trade comprehensive coverage for high resolution and are subject to the inherent biases of RNA and protein capture from a 2D slice of a dynamic 3D process. Tissue handling, fixation, and perioperative timing can introduce artifacts that may be mistaken for biological signals. Therefore, improving reproducibility will require robust cross-platform integration, batch-aware computational analysis, and the transparent preregistration of spatial endpoints. Mechanistically, it remains critical to determine which features are foundational to niche formation versus those that merely maintain it, and how factors such as brain region, patient age, and comorbidities influence these spatial dynamics.

## 5 Conclusion

In summary, based on the current findings, we hypothesize that epilepsy is not merely an inflammatory disease of the brain but a disease of inflammatory places in the brain. By moving from generic neuroinflammation to mapped immune microenvironments, we will gain an explanation for where seizures begin, why they persist despite standard therapy, and how to target them with greater precision. The practical path forward pairs spatial biomarker-guided stratification with interventions that repair the local infrastructure of stability. If we can collapse the niche, we may finally bend epilepsy toward disease modification rather than perpetual symptom control.
